# Using 5 consecutive years of NICE guidance to describe the characteristics and influencing factors on the economic evaluation of orphan oncology drugs

**DOI:** 10.3389/fpubh.2022.964040

**Published:** 2022-09-14

**Authors:** Duan Shengnan, Lv Zixuan, Zhou Na, Zhu Weikai, Yi Yuanyuan, Liu Jiasu, Yuan Ni

**Affiliations:** ^1^School of Public Health, Dalian Medical University, Dalian, China; ^2^Department of Health Policy and Management, School of Public Health, Peking University, Beijing, China; ^3^Department of Traditional Chinese Medicine, The First Affiliated Hospital of Dalian Medical University, Dalian, China; ^4^Department of Respiratory Medicine, The First Affiliated Hospital of Dalian Medical University, Dalian, China; ^5^Department of Ophthalmology, The Second Affiliated Hospital of Dalian Medical University, Dalian, China

**Keywords:** orphan oncology drugs, NICE, economic evaluation, influencing factors, technology appraisal guidance

## Abstract

**Objective:**

Orphan oncology drugs used in this article were defined by the type of disease treated by drugs, as drugs used to treat rare diseases with a prevalence of ≤ 500 per million people per year. In this article, our concern was to explore focus on the economic evaluation of the National Institute for Health and Care Excellence (NICE), when orphan oncology drugs were appraised for reimbursement, and provide advice and suggestions to decision-makers.

**Methods:**

A retrospective study was used in this study. Thirty guidance were gathered as our subject by NICE from 2016 to 2020, excluded drugs were not identified as orphan by European Medicines Agency (EMA) and orphan drugs were not used for cancer, and orphan oncology drugs were terminated at the time of data collection at NICE. Qualitative analysis, descriptive statistics, and Fisher's exact test were conducted.

**Results:**

Of all guidance, the partitioned survival model was used most to appraise orphan oncology drugs, and every drug had a kind of commercial arrangement such as patient access scheme (PAS), managed access arrangements (MAAs), and commercial access agreement (CAAs). End of life is an important indicator that had been defined by NICE in the methods of technology appraisal in 2013, and drugs that met the criterion would be given a higher threshold of ICER. In addition, we found that potential health benefits were increasingly concerned such as drug delivery.

**Conclusion:**

In the setting of uncertain clinical and cost efficacy, orphan oncology drugs are comprehensively evaluated in multiple additional dimensions, which include life-extending benefits, and innovation. NICE uses a combination of special considerations for incomplete data, appropriate economic models, and appropriate health technology assessment (HTA) methods during the assessment process, besides, orphan oncology drugs with insufficiency evidence were recommended Cancer Drugs fund (CDF) to afford for patients, which would obtain more availability and accessibility, based on which, high-quality drugs for treating rare cancers can fall within the scope of affordable healthcare provided by the English medical insurance fund.

## Introduction

Rare diseases are also known as Orphan diseases, which were used to refer to some uncommon, low incidence, and often life-threatening diseases. Drugs that treat orphan diseases are called orphan drugs. Orphan drugs, or Orphan Medicinal Products (OMPs), exist for <3% of rare diseases (1). It is shown that the number of orphan drug designations assigned by the United States Food and Drug Administration (FDA) significantly increased from 1983 to 2019, most prominently in oncology (1910, 37%) (2). Another study on orphan drug approvals in Europe found that 39% of all orphan drugs that were approved by the European Medicines Agency through a centralized process were cancer-related (3). Nine of the top 10 indications for orphan drugs as specified by the European Medicines Agency/European Mes Aedicingency (EMA) were for cancer (including acute myeloid leukemia, non-Hodgkin's lymphoma, glioma, pancreatic cancer, ovarian cancer, multiple myeloma, renal cell carcinoma, liver cancer, and chronic lymphocytic leukemia) (4).

There were two definitions of orphan drugs used in oncology. Take the European Union as an example, one is defined by the type of disease treated by drugs, such as orphan drugs in oncology, oncology drugs with orphan designation, medicines for orphan indications in oncology, oncology Orphan drugs (ODs), which were defined as drugs used to treat rare diseases with a prevalence ≤ 500 per million people per year. They are usually referred to by the following terms: orphan drugs in oncology, oncology drugs with an orphan designation, medicines for orphan indications in oncology, and oncology ODs. This article focuses on this definition, using “orphan oncology drugs.” An alternative definition defines orphan drugs as those used to treat cancers with an incidence of ≤ 60 per million people/year, and refers to them as rare tumor drugs. There are about 200 rare tumors in Europe, which account for 20–24% of all tumor diagnoses (5). These drugs are usually referred to by one of the following terms: rare cancers, rare tumors, rare neoplastic disorders, and oncology drugs in the treatment of rare diseases.

Providing equal access to affordable drugs across countries is high on the political agenda in many countries, even though it is far from being achieved (6). Consequently, all countries are exploring ways and methods that suit their own country to provide access to affordable drugs, and some have already had special and well-established HTA agencies (7), such as the National Institute for Health and Care Excellence (NICE) in the UK, the Scottish Medicines Consortium (SMC) in Scotland (8), the Dental and Pharmaceutical Benefits Board (TLV) in Sweden and the Haute Autorité de santé (HAS) in France (9).

There is still a big distance in the average number between non-orphans and orphan drugs (10). Even though rare disease has a low incidence, drugs for rare diseases offer important health benefits and continue to challenge traditional health technology assessment (HTA) (11). But on account of the high treatment cost, a small number of subjects, uncertain clinical effects, social value, and other problems, it is difficult to appraise orphan oncology drugs, and conventional appraisal methods were not applicable anymore. It is necessary to explore multiple appraisal methods when evaluating orphan oncology drugs, including the use of alternative indicators, incomplete data processing, and social value factors.

National Institute for Health and Care Excellence/NICE in the UK has a separate process through which certain drugs for rare diseases are reviewed from the outset (12), which made an important role in producing evidence-based guidance and advice for health, public health, and social care practitioners. They did technology appraisal guidance (TAG, beginning in 2000) and highly specialized technologies (HST, beginning in 2015, which was only used to consider drugs for very rare conditions) to make recommendations on the clinical and cost-effectiveness of drugs (13), which were used to help to ensure that the NHS uses its resources fairly and effectively. Drugs were appraised based on a review of clinical and economic evidence. And there are 5 types (recommended, optimized, Cancer Drugs Fund, not recommended, only in research) of recommendations after appraisal, which they can make. Appraisal recommendations are prepared by independent committees, which provides a good reference for us to appraise orphan oncology drugs.

This study evaluates the guidance of orphan oncology drugs appraised by NICE from 2016 to 2020 and focuses on the economic assessment of these drugs in order to explore concerns on the economic evaluation of NICE when orphan oncology drugs were appraised for reimbursement, which was aimed to generalize findings and provide advice and suggestions to decision-makers.

## Materials and methods

### Sampling and inclusion criteria for HTA agencies and drugs

We conducted a retrospective study on guidance published on the website of the National Institute for Health and Care Excellence/NICE from 2016 to 2020 (https://www.nice.org.uk/guidance/published?ndt=Guidance&ndt=Quality%20standard.NICE) was chosen for four reasons: (i) it is well-established, (ii) its guidance had been publicly available, (iii) it made a very important role in the final reimbursement decision in the UK, (iv) and its guidance had been reported in a language understood.

For the included drugs, we first collected drug guidance from the guidance program of highly specialized technologies (HST) and technology appraisal guidance (TAG) (14) on the website of NICE. Second, Human medicine European public assessment reports on the European Medicines Agency (EMA) webpage (15) were used to identify if drugs were designated as orphan drugs and authorized for use in the European Union. There were 286 drugs that have been published from 2016 to 2020, including 11 from the HST guidance program and 275 from the TAG program, 62 of which received an orphan EMA designation. Those were excluded because indications were not on oncology and appraisal were terminated at the time of data collection at NICE. Finally, a total of 30 guidance of orphan oncology drugs were selected (Table 1).

**Table 1:** Orphan oncology drugs guidance from 2016 to 2020.

**Year**	**Guidance no. of HTG and TAG**	**Guidance no. of orphan drugs**	**Guidance number of orphan oncology drugs**			
			**Total**	**Recommend to NHS**	**Recommend to CDF**	**Not recommend**
2016	53	2	1	1	0	0
2017	63	12	7	5	0	2
2018	57	17	15	8	6	1
2019	60	18	8	2	3	3
2020	53	13	7	5	0	2
**Total**	**286**	**62**	**38**	**21**	**9**	**8**

### Study design and methodological framework

**Figure 1:**
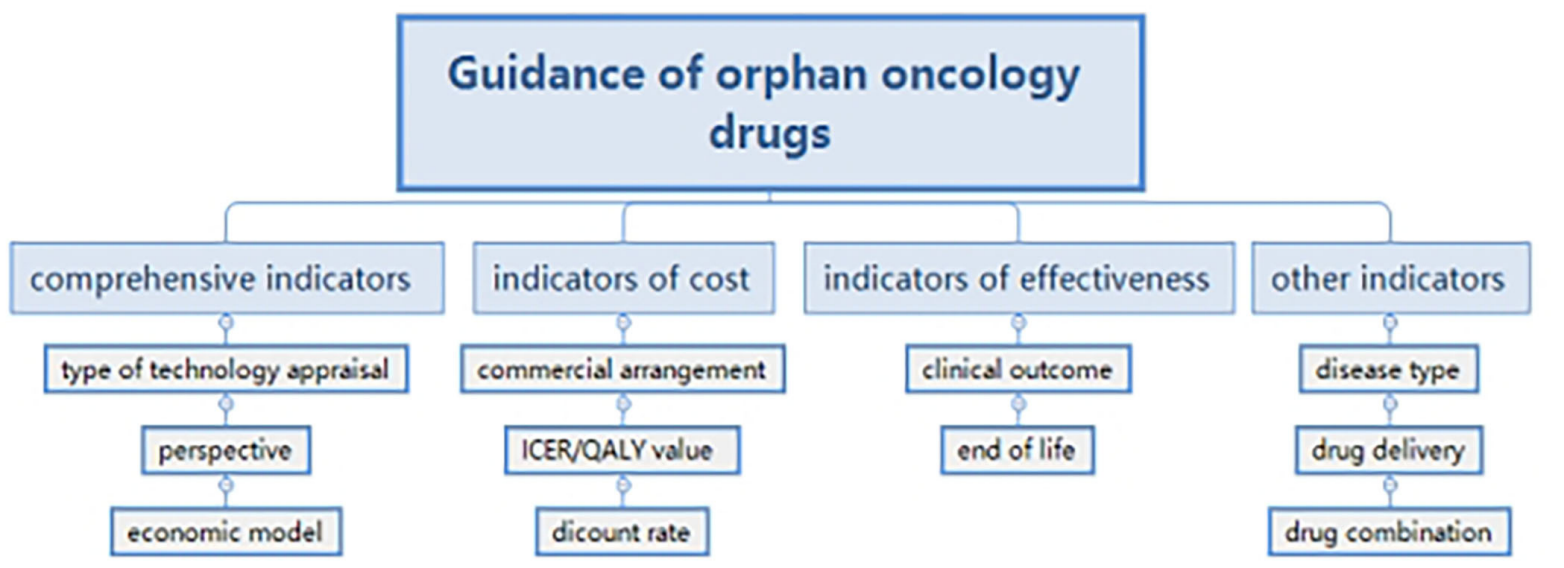
Methodological framework.

We paid attention to the economic analysis of guidance and formulated a framework with the method of thematic analysis (16). Indicators in guidance were extracted and classified into four groups. Comprehensive indicators included: technology appraisal; perspective and economic model; indicators of cost such as a commercial arrangement; Incremental cost effectiveness ratio (ICER)/QALY (quality-adjusted life year) value and discount rate; indicators of effectiveness, such as clinical outcome and end of life; and other indicators such as disease type, drug delivery, and drug combination (Figure 1). Because of the qualitative decisions (17), the research did not aim at quantitative indicators, but to generalize findings and supply suggestions for the government.

## Results

There were 30 orphan oncology drug guidance between 2016 to 2020 on the NICE website were chosen in this study. And among the 30 orphan oncology drugs, 21 were recommended to NHS, and 9 were recommended to CDF. The content of guidance of 30 orphan oncology drugs would be described in four sections as the framework.

### Comprehensive indicators

**Figure 2:**
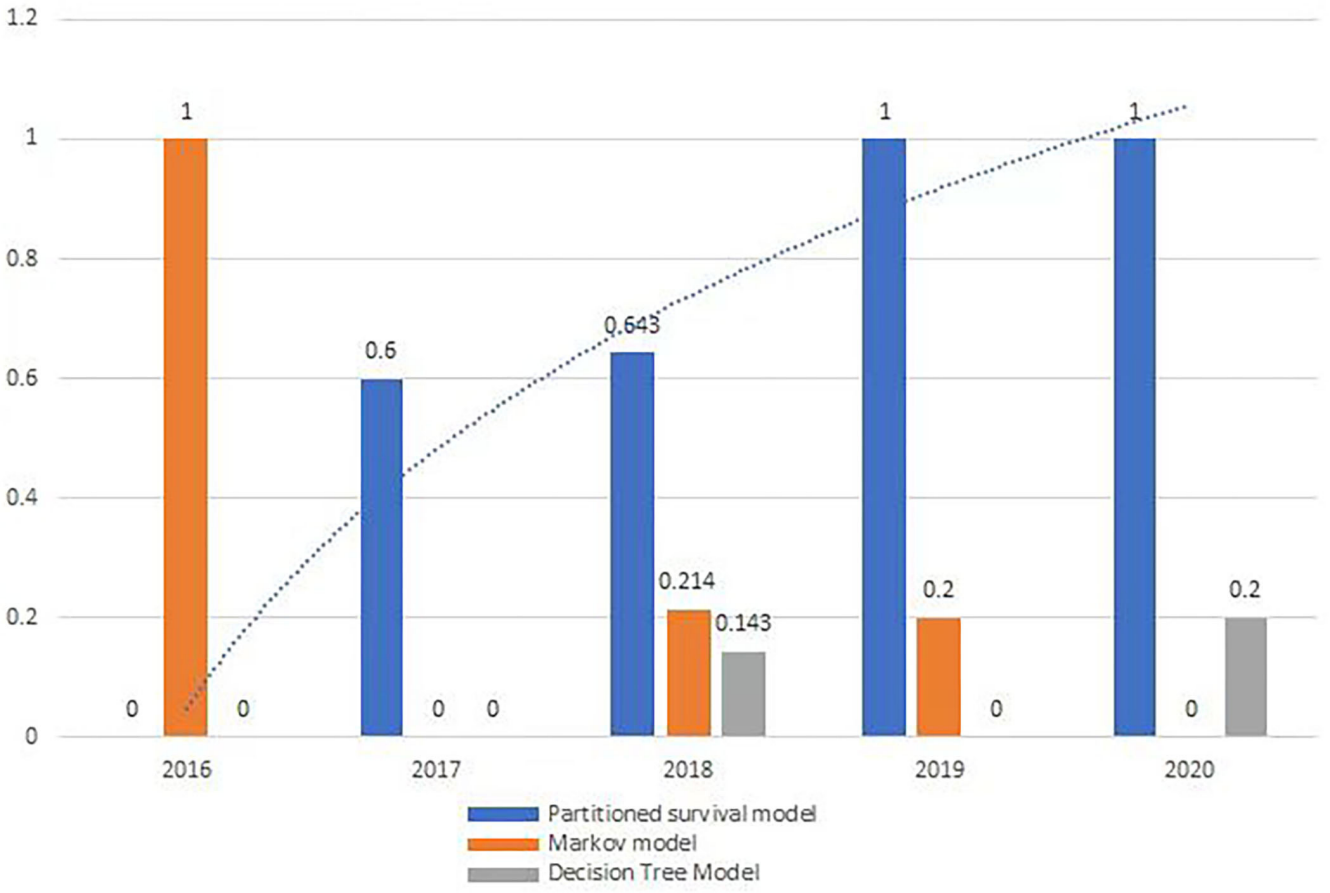
The usage of partition survival model.

From 2016 to 2020, all orphan oncology drugs were appraised by Single Technology Appraisal (STA), except in 2018. There was a growing trend in the usage of partition survival models (Figure 2), but no significant trend on indicators of comprehensive indicators.

#### Type of technology appraisal

Of the 30 recommended orphan oncology drugs, 27 (90%) were appraised *via* Single Technology Appraisal (STA) and 3 (10%) were appraised *via* Multiple Technology Appraisal (MTA). A single technology appraisal (STA) covers a single technology for a single indication. A multiple technology appraisal (MTA) normally covers more than one technology or one technology for more than one indication. The three orphan oncology drugs appraised by MTA were lutetium (177Lu) oxodotreotide for treating unresectable or metastatic neuroendocrine tumors, cabozantinib for treating medullary thyroid cancer, and lenvatinib and sorafenib for treating differentiated thyroid cancer after radioactive iodine, all of whose guidance was published in 2018 (Table 2).

**Table 2:** Details of comprehensive indicators in guidance from 2016 to 2020.

**Indicators** ***No.(%)***	**Total condition from 2016 to 2020**			**2016**			**2017**			**2018**			**2019**			**2020**		
	**To NHS**	**To CDF**	**Total**	**To NHS**	**To CDF**	**Total**	**To NHS**	**To CDF**	**Total**	**To NHS**	**To CDF**	**Total**	**To NHS**	**To CDF**	**Total**	**To NHS**	**To CDF**	**Total**
***Comprehensive indicators***																		
**Type of technology appraisal**																		
Single technology appraisal/STA	18(60%)	9(30%)	27(90%)	1(100%)	0(0%)	1(100%)	5(100%)	0(0%)	5(100%)	5(35.7%)	6(42.9%)	11(78.6%)	2(40%)	3(60%)	5(100%)	5(100%)	0(0%)	5(100%)
Multiple technology appraisal/MTA	3(10%)	0(0%)	3(10%)	0(0%)	0(0%)	0(0%)	0(0%)	0(0%)	0(0%)	3(21.4%)	0(0%)	3(21.4%)	0(0%)	0(0%)	0(0%)	0(0%)	0(0%)	0(0%)
**Perspective**																		
National health service/NHS	3(10%)	2(6.7%)	5(16.7%)	0(0%)	0(0%)	0(0%)	1(20%)	0(0%)	1(20%)	0(0%)	1(7.1%)	1(7.1%)	2(40%)	1(20%)	3(60%)	0(0%)	0(0%)	0(0%)
NHS and PSS	5(16.7%)	3(10%)	8(26.7%)	1(100%)	0(0%)	1(100%)	2(40%)	0(0%)	2(40%)	0(0%)	1(7.1%)	1(7.1%)	0(0%)	2(40%)	2(40%)	2(40%)	0(0%)	2(40%)
Unspecified	13(43.3%)	4(13.3%)	16(53.3%)	0(0%)	0(0%)	0(0%)	2(40%)	0(0%)	2(40%)	7(50%)	4(28.6%)	11(78.6%)	0(0%)	0(0%)	0(0%)	3(60%)	0(0%)	3(60%)
**Economic model**																		
Partitioned survival model	15(50%)	7(23.3%)	22(73.3%)	0(0%)	0(0%)	0(0%)	3(60%)	0(0%)	3(60%)	5(35.7%)	4(28.6%)	9(64.3%)	2(40%)	3(60%)	5(100%)	5(100%)	0(0%)	5(100%)
Markov model	4(13.3%)	1(3.3%)	5(16.7%)	1(100%)	0(0%)	1(100%)	0(0%)	0(0%)	0(0%)	2(14.3%)	1(7.1%)	3(21.4%)	1(20%)	0(0%)	1(20%)	0(0%)	0(0%)	0(0%)
Decision tree model	1(3.3%)	2(6.7%)	3(10%)	0(0%)	0(0%)	0(0%)	0(0%)	0(0%)	0(0%)	0(0%)	2(14.3%)	2(14.3%)	0(0%)	0(0%)	0(0%)	1(20%)	0(0%)	1(20%)
Unspecified	3(10%)	1(3.3%)	4(13.3%)	0(0%)	0(0%)	0(0%)	2(40%)	0(0%)	2(40%)	1(7.1%)	1(7.1%)	2(14.3%)	0(0%)	0(0%)	0(0%)	0(0%)	0(0%)	0(0%)

#### Perspective

Of the guidance that presented a specific and clear perspective, 8 (26.7%) analyses were conducted from the perspective of the NHS and Personal Social Services (PSS), and 5 (16.7%) from the perspective of the NHS without PSS. A total of 16 drugs did not formulate a perspective in their guidance (Table 2). The scope described by NICE usually advocates that the company or ERG should provide an NHS and PSS perspective to study the drug. However, because of the small number of patients with a specific orphan disease, it is difficult to perform a clinical trial. The company or ERG therefore only did their study from the NHS or payer perspective.

### Data analysis

Qualitative analysis was used in the first stage of the research. On the basis of the framework, all the relevant information at each step of the decision process was identified. Then, the data collected was exported into excel for analysis. Descriptive statistics were conducted to determine the types and frequencies of indicators. A Fisher's exact test was used to measure associations between recommendation and ICER/end of life/drug delivery/drug combination.

#### Economic model

From 2016 to 2020, a partitioned survival model was used to perform a cost-effectiveness analysis for 22 orphan oncology drugs (73.3%), a Markov model for 5 (16.7%), and a decision tree model for 3 (10%). Four orphan oncology drugs did not document the type of economic model used in their guidance (Table 2).

Several economic models were combined for the cost-effectiveness analysis of four drugs. The economic model for Pomalidomide, a treatment for multiple myeloma that was previously combined with lenalidomide and bortezomib, in 2017 was a semi-Markov partitioned survival structure. In 2018, Tisagenlecleucel was evaluated using a partitioned-survival model, semi-Markov, and decision tree model. Blinatumomab, which was appraised in 2019 for treating acute lymphoblastic leukemia in remission with minimal residual disease activity, was analyzed using a partitioned-survival model and a semi-Markov model. Gilteritinib, which was appraised in 2020 as a treatment for relapsed or refractory acute myeloid leukemia, was assessed using a decision-tree structure followed by partitioned survival models.

### Indicators of cost

#### Commercial arrangement

All 30 orphan oncology drugs have commercial arrangements. A total of 21(70%) had a patient access scheme (PAS) *via* a simple discount, 7 (23.3%) had a managed access arrangement (MAA), and 2 (6.7%) had a commercial access agreement (CAA) (Table 3).

**Table 3:** Details of cost indicators in guidance from 2016 to 2020.

**Indicators** ***No.(%)***	**Total condition from 2016 to 2020**			**2016**			**2017**			**2018**			**2019**			**2020**		
	**To NHS**	**To CDF**	**Total**	**To NHS**	**To CDF**	**Total**	**To NHS**	**To CDF**	**Total**	**To NHS**	**To CDF**	**Total**	**To NHS**	**To CDF**	**Total**	**To NHS**	**To CDF**	**Total**
***Cost indicators***																		
**Commercial arrangement**																		
Patient access schemes/PAS	19(63.3%)	2(6.7%)	21(70%)	1(100%)	0(0%)	1(100%)	4(80%)	0(0%)	4(80%)	7(50%)	2(6.7%)	9(30%)	2(40%)	0(0%)	2(40%)	5(100%)	0(0%)	5(100%)
Commercial access agreement/CAA	2(6.7%)	0(0%)	2(6.7%)	0(0%)	0(0%)	0(0%)	1(20%)	0(0%)	1(20%)	1(7.1%)	0(0%)	1(7.1%)	0(0%)	0(0%)	0(0%)	0(0%)	0(0%)	0(0%)
Managed access arrangement /MAAs (Contains PAS, CAA)	0(0%)	7(23.3%)	7(23.3%)	0(0%)	0(0%)	0(0%)	0(0%)	0(0%)	0(0%)	0(0%)	4(28.6%)	4(28.6%)	0(0%)	3(60%)	3(60%)	0(0%)	0(0%)	0(0%)
**ICER/QALY value**																		
≤ £20,000	1(3.3%)	1(3.3%)	2(6.7%)	0(0%)	0(0%)	0(0%)	0(0%)	0(0%)	0(0%)	0(0%)	1(7.1%)	1(7.1%)	0(0%)	0(0%)	0(0%)	1(20%)	0(0%)	1(20%)
£20,000-£30,000	10(33.3%)	1(3.3%)	11(36.7%)	1(100%)	0(0%)	1(100%)	1(20%)	0(0%)	1(20%)	5(35.7%)	1(7.1%)	6(42.9%)	2(40%)	0(0%)	2(40%)	1(20%)	0(0%)	1(20%)
≥£30,000	10(33.3%)	6(20%)	16(53.3%)	0(0%)	0(0%)	0(0%)	4(80%)	0(0%)	4(80%)	3(21.4%)	3(21.4%)	6(42.9%)	0(0%)	3(60%)	3(60%)	3(60%)	0(0%)	3(60%)
Unspecified	0(0%)	1(3.3%)	1(3.3%)	0(0%)	0(0%)	0(0%)	0(0%)	0(0%)	0(0%)	0(0%)	1(7.1%)	1(7.1%)	0(0%)	0(0%)	0(0%)	0(0%)	0(0%)	0(0%)
**Discount rate**																		
1.50%	2(6.7%)	1(3.3%)	3(10%)	1(100%)	0(0%)	1(100%)	0(0%)	0(0%)	0(0%)	1(7.1%)	1(7.1%)	2(14.3%)	0(0%)	0(0%)	0(0%)	0(0%)	0(0%)	0(0%)
3.50%	8(26.7)	4(13.3%)	12(40%)	0(0%)	0(0%)	0(0%)	3(60%)	0(0%)	3(60%)	1(7.1%)	1(7.1%)	2(14.3%)	1(20%)	3(60%)	4(80%)	3(60%)	0(0%)	3(60%)
Unpublished	11(36.7%)	4(13.3%)	15(50%)	0(0%)	0(0%)	0(0%)	2(40%)	0(0%)	2(40%)	6(35.7%)	4(28.6%)	10(71.4%)	1(20%)	0(0%)	1(20%)	2(40%)	0(0%)	2(40%)

#### ICER/QALY value

The ICER/QALY value of 2 orphan oncology drugs (6.7%) was below £20,000, 11 orphan oncology drugs (36.7%) were between £20,000 and £30,000, while that of 16 orphan oncology drugs (53.3%) was above £30,000. One drug's ICER/QALY value was not published. And there was no significant difference between ICER/QALY value and recommendations (*p* = 0.238) (Table 3).

#### Discount rate

The discount rate of pharmaceuticals is generally between 3 and 5% (18). The discount rates for orphan oncology drugs from 2016 to 2020 were 1.5% (10% of drugs) and 3.5% (40% of drugs), which are the same as the UK standard obtained in ISPOR (19). The discount rates of 15 orphan oncology drugs were not published because of commercial privacy (Table 3).

### Indicators of effectiveness

#### Clinical outcome

The most common clinical indicator was overall survival (OS), which was included in the economic models of 29 of the 30 recommended orphan oncology drugs. The second most frequent was progression-free survival (PFS), which was included in 83.3%. Other indicators included complete response (CR, 30%), response rates (RR, 26.7%), and event-free survival (EFS, 20%). Less common indicators included objective response rate (ORR, 10%), disease-free survival (DFS, 10%), time to next treatment (10%), relapse-free survival (RFS, 6.7%), overall remission rate (ORR, 6.7%), minimal residual disease (MRD, 6.7%), and duration of response (DoR, 6.7%). The clinical outcome indicator of response time was used only once in 2016 for Panobinostat (Table 4).

**Table 4:** Details of indicators of effectiveness in guidance from 2016 to 2020.

**Indicators** ***No.(%)***	**Total condition from 2016 to 2020**			**2016**			**2017**			**2018**			**2019**			**2020**		
	**To NHS**	**To CDF**	**Total**	**To NHS**	**To CDF**	**Total**	**To NHS**	**To CDF**	**Total**	**To NHS**	**To CDF**	**Total**	**To NHS**	**To CDF**	**Total**	**To NHS**	**To CDF**	**Total**
***Indicators of effectiveness***																		
**Clinical outcome**																		
Overall survival/OS	20(66.7%)	9(30%)	29(96.7%)	1(100%)	0(0%)	1(100%)	5(100%)	0(0%)	5(100%)	8(100%)	6(42.9%)	14(100%)	1(20%)	3(60%)	4(80%)	5(100%)	0(0%)	5(100%)
Progression-free survival/PFS	16(53.3%)	9(30%)	25(83.3%)	1(100%)	0(0%)	1(100%)	4(80%)	0(0%)	4(80%)	5(35.7%)	6(42.9%)	11(78.6%)	2(40%)	3(60%)	5(100%)	4(80%)	0(0%)	4(80%)
Objective response rate/ORR	2(6.7%)	1(3.3%)	3(10%)	0(0%)	0(0%)	0(0%)	1(20%)	0(0%)	1(20%)	0(0%)	1(7.1%)	1(7.1%)	1(20%)	0(0%)	1(20%)	0(0%)	0(0%)	0(0%)
Complete response/CR	7(23.3%)	2(6.7%)	9(30%)	0(0%)	0(0%)	0(0%)	2(40%)	0(0%)	2(40%)	1(7.1%)	2(14.3%)	3(21.4%)	1(20%)	0(0%)	1(20%)	3(60%)	0(0%)	3(60%)
Relapse-free survival/RFS	1(3.3%)	1(3.3%)	2(6.7%)	0(0%)	0(0%)	0(0%)	1(20%)	0(0%)	1(20%)	0(0%)	1(7.1%)	1(7.1%)	0(0%)	0(0%)	0(0%)	0(0%)	0(0%)	0(0%)
Disease-free survival/DFS	3(10%)	0(0%)	3(10%)	0(0%)	0(0%)	0(0%)	0(0%)	0(0%)	0(0%)	3(21.4%)	0(0%)	3(21.4%)	0(0%)	0(0%)	0(0%)	0(0%)	0(0%)	0(0%)
Event-free survival /EFS	5(16.7%)	1(3.3%)	6(20%)	0(0%)	0(0%)	0(0%)	1(20%)	0(0%)	1(20%)	3(21.4%)	1(7.1%)	4(28.6%)	0(0%)	0(0%)	0(0%)	1(20%)	0(0%)	1(20%)
Overall remission rate/ORR	2(6.7%)	0(0%)	2(6.7%)	0(0%)	0(0%)	0(0%)	0(0%)	0(0%)	0(0%)	1(7.1%)	0(0%)	1(7.1%)	0(0%)	2(40%)	2(40%)	1(20%)	0(0%)	1(20%)
Minimal residual disease/MRD	1(3.3%)	1(3.3%)	2(6.7%)	0(0%)	0(0%)	0(0%)	0(0%)	0(0%)	0(0%)	0(0%)	1(7.1%)	1(7.1%)	1(20%)	1(20%)	2(40%)	0(0%)	0(0%)	0(0%)
Response rates/RR	5(16.7%)	3(10%)	8(26.7%)	1(100%)	0(0%)	1(100%)	2(40%)	0(0%)	2(40%)	2(14.3%)	2(14.3%)	4(28.6%)	0(0%)	1(20%)	1(20%)	0(0%)	0(0%)	0(0%)
Duration Of response/DoR	2(6.7%)	0(0%)	2(6.7%)	0(0%)	0(0%)	0(0%)	1(20%)	0(0%)	1(20%)	0(0%)	0(0%)	0(0%)	0(0%)	0(0%)	0(0%)	1(20%)	0(0%)	1(20%)
Time to next treatment	1(3.3%)	2(6.7%)	3(10%)	1(100%)	0(0%)	1(100%)	0(0%)	0(0%)	0(0%)	0(0%)	2(14.3%)	2(14.3%)	0(0%)	0(0%)	0(0%)	0(0%)	0(0%)	0(0%)
Time to response	1(3.3%)	0(0%)	1(3.3%)	0(0%)	0(0%)	0(0%)	1(20%)	0(0%)	1(20%)	0(0%)	0(0%)	0(0%)	0(0%)	0(0%)	0(0%)	0(0%)	0(0%)	0(0%)
**End-of-life**																		
Meet criterion, sufficient evidence	7(23.3%)	3(10%)	10(33.3%)	0(0%)	0(0%)	0(0%)	4(80%)	0(0%)	3(60%)	2(14.3%)	1(7.1%)	3(21.4%)	0(0%)	2(40%)	2(40%)	1(20%)	0(0%)	2(40%)
Meet criterion, insufficient evidence	6(20.0%)	0(0%)	6(20%)	0(0%)	0(0%)	0(0%)	1(20%)	0(0%)	1(20%)	3(21.4%)	0(0%)	3(21.4%)	0(0%)	0(0%)	0(0%)	2(40%)	0(0%)	1(20%)
Not meet criterion	7(23.3%)	5(16.7%)	12(40%)	1(100%)	0(0%)	1(100%)	0(0%)	0(0%)	0(0%)	3(21.4%)	4(28.6%)	7(50%)	2(40%)	1(20%)	3(60%)	1(20%)	0(0%)	1(20%)
Unspecified	1(3.3%)	1(3.3%)	2(6.7%)	0(0%)	0(0%)	0(0%)	0(0%)	0(0%)	1(20%)	0(0%)	1(7.1%)	1(7.1%)	0(0%)	0(0%)	0(0%)	1(20%)	0(0%)	1(20%)

#### End of life

The majority of the published guidance on drugs in our study (28, accounting for 93.3%) included a separate paragraph to discuss evidence regarding end-of-life and orphan oncology drugs. A total of 10 orphan oncology drugs met NICE's criteria for being considered a life-extending treatment and had sufficient evidence, 12 orphan oncology drugs did not meet NICE's criteria, and 6 orphan oncology drugs were considered a life-extending treatment but had uncertain cost-effectiveness estimates. 2 orphan oncology drugs had unspecified end-of-life guidance (Table 4).

### Other indicators

#### Disease type and indication

It is shown that 80% of indications of disease were blood and bone marrow cancers, including multiple myeloma, large cell lymphoma, Philadelphia chromosome-negative acute lymphoblastic leukemia, acute lymphoblastic leukemia (ALL), acute myeloid leukemia (AML), CD30-positive Hodgkin lymphoma, follicular lymphoma, large B-cell lymphoma, and chronic lymphocytic leukemia (CLL), followed by thyroid cancer (6.7%), which included medullary thyroid cancer. liver cancers, metastases, head and neck cancers, and ovarian cancer were the target for 3.3% of treatment each (Table 5).

**Table 5:** Details of indicators of effectiveness in guidance from 2016 to 2020.

**Indicators** ***No.(%)***	**Total condition from 2016 to 2020**			**2016**			**2017**			**2018**			**2019**			**2020**		
	**To NHS**	**To CDF**	**Total**	**To NHS**	**To CDF**	**Total**	**To NHS**	**To CDF**	**Total**	**To NHS**	**To CDF**	**Total**	**To NHS**	**To CDF**	**Total**	**To NHS**	**To CDF**	**Total**
***Orter indicators***																		
**Disease type**																		
Blood and bone marrow cancers	17(56.7%)	7(23.3%)	24(80%)	1(100%)	0(0%)	1(100%)	4(80%)	0(0%)	4(80%)	5(35.7%)	4(28.6%)	9(64.3%)	2(40%)	3(60%)	5(100%)	5(100%)	0(0%)	5(100%)
Liver cancers	1(3.3%)	0(0%)	1(3.3%)	0(0%)	0(0%)	0(0%)	1(20%)	0(0%)	1(20%)	0(0%)	0(0%)	0(0%)	0(0%)	0(0%)	0(0%)	0(0%)	0(0%)	0(0%)
Head and neck cancers	0(0%)	1(3.3%)	1(3.3%)	0(0%)	0(0%)	0(0%)	0(0%)	0(0%)	0(0%)	0(0%)	1(7.1%)	1(7.1%%)	0(0%)	0(0%)	0(0%)	0(0%)	0(0%)	0(0%)
Metastases	1(3.3%)	0(0%)	1(3.3%)	0(0%)	0(0%)	0(0%)	0(0%)	0(0%)	0(0%)	1(7.1%)	0(0%)	1(7.1%)	0(0%)	0(0%)	0(0%)	0(0%)	0(0%)	0(0%)
Ovarian cancer	0(0%)	1(3.3%)	1(3.3%)	0(0%)	0(0%)	0(0%)	0(0%)	0(0%)	0(0%)	0(0%)	1(7.1%)	1(7.1%%)	0(0%)	0(0%)	0(0%)	0(0%)	0(0%)	0(0%)
Thyroid cancer	2(6.7%)	0(0%)	2(6.7%)	0(0%)	0(0%)	0(0%)	0(0%)	0(0%)	0(0%)	2(6.7%)	0(0%)	2(6.7%)	0(0%)	0(0%)	0(0%)	0(0%)	0(0%)	0(0%)
**Drug delivery**																		
Oral	8(26.7%)	2(6.7%)	10(33.3%)	1(100%)	0(0%)	1(100%)	3(60%)	0(0%)	3(60%)	3(21.4%)	2(14.3%)	5(35.7%)	0(0%)	0(0%)	0(0%)	1(20%)	0(0%)	1(20%)
Infusion	13(43.3%)	7(23.3%)	20(66.7%)	0(0%)	0(0%)	0(0%)	2(40%)	0(0%)	2(40%)	5(35.7%)	4(28.6%)	9(64.3%)	2(40%)	3(60%)	5(100%)	4(80%)	0(0%)	4(80%)
**Drug combination**																		
Yes	8(26.7%)	2(6.7%)	10(33.3%)	1(100%)	0(0%)	1(100%)	1(20%)	0(0%)	1(20%)	1(7.1%)	2(14.3%)	3(21.4%)	1(20%)	0(0%)	1(20%)	4(80%)	0(0%)	4(80%)
No	13(43.3%)	7(23.3%)	20(66.7%)	0(0%)	0(0%)	0(0%)	4(80%)	0(0%)	4(80%)	7(50%)	4(28.6%)	11(78.6%)	1(20%)	3(60%)	4(80%)	1(20%)	0(0%)	1(20%)

Of all the 30 drugs, 4 (13.3%) were used for treating diseases at least 2 previous treatments, 8 (26.7%) were used for relapsed or refractory disease, 9 (30%) were used for treating acute disease, and 4 (13.3%) were used for treating untreated disease. Most drugs were used for adults, except Kymriah (Tisagenlecleucel), which was used to treat relapsed or refractory B-cell acute lymphoblastic leukemia in people aged up to 25 years.

#### Drug delivery

Of all of the recommended drugs, 33.3% were taken orally and the rest (66.7%) were administered by intravenous infusion. And the difference between drug delivery and recommendation found has no significance (*p* = 0.675) (Table 5).

#### Drug combination

Drug combinations were recommended for 33.3% of orphan drugs. Daratumumab, which is used for CD30-positive cutaneous T-cell lymphoma, was recommended as a treatment option after the failure of at least one systemic therapy in adults when combined with bortezomib and dexamethasone. Carfilzomib, used for treating multiple myeloma in adults, was only recommended following the failure of at least one systemic therapy when combined with either lenalidomide and dexamethasone or dexamethasone alone. Polatuzumab vedotin was indicated for treating relapsed or refractory diffuse large B-cell lymphoma when combined with rituximab and bendamustine. There were no significant differences between drug combination and recommendation (*p* = 0.675) (Table 5).

## Discussion

### Drugs recommended to CDF may obtain more availability and accessibility

Orphan oncology drugs can be recommended to the NHS or CDF. The guidance of the drugs that were recommended to the NHS will be reviewed after 2 or 3 years. Drugs were usually recommended for use as an option for the Cancer Drugs Fund because of uncertain cost-effectiveness estimates, immature survival data, an uncertain impact of the treatment on patient life expectancy, and in incomplete compliance with end-of-life standards. Drugs recommended to the CDF would not impede patient use while more evidence is collected for a final NICE review and a final recommendation regarding NHS use. To some degree, orphan oncology drugs recommended to CDF may obtain more availability and accessibility, a study showed that there is greater availability and accessibility of orphan medicines in England where most of the 68 OMPs were reimbursed because they were included in the NHS England specialized commissioning list [32] or the Cancer Drugs Fund [13], compared with Scotland and Wales (20). This kind of dynamic management mechanism is important to learn.

### Partitioned survival model was increasingly used to appraise orphan oncology drugs

The most useful economic model for orphan oncology drugs in all the guidance was the partitioned survival model, and there was an increasing trend from 2016 to 2020 (Figure 2), which is in line with the work by Williams et al. (21) and others (22, 23). Partition survival models are often used in the economic evaluation of drugs in oncology mostly depending on the fact that it does not have to calculate the metastatic probability of the disease, and also do not require a large number of model assumptions, closer to the actual observed data; The survival curve can be directly applied to obtain the proportion of patients with different health statuses (22), and the complex risk function can be directly reconstructed by extrapolation (23).

### Patient access schemes (PAS) and CAAs were broadly used to control the price, and MAAs were used to provide a vital alternative route for patients to access these treatments

Every orphan oncology drug has at least one commercial arrangement. Drugs recommended to the NHS usually have PAS, which entails a simple discount. However, it is a pity that we cannot ascertain the amount that was discounted due to commercial confidentiality.

Managed access arrangements (MAAs) are an agreement between NHS England and a company. Some drugs cannot be recommended due to uncertainty about their value for money, MAAs were used by the time to collect more evidence to address the uncertainties. Usually, without managed access, NICE might not be able to recommend patients have access to these promising new drugs at that time. Managed access provides a vital alternative route for patients to access these treatments. In our study, MAAs were all used for drugs recommended to the CDF, which include a Data Collection Agreement (DCA), and Commercial Access Agreement (CAA), with a simple discount PAS. However, CAAs were also unpublished because of commercial confidentiality. Drugs were funded through the CDF for a limited period of time (up to 2 years), during which MAAs will be maintained in accordance with (1) the results that need to be collected to address uncertainty in key clinical areas, and (2) the cost of the drug regulatory access agreement. The drug will then undergo rapid reconsideration to decide if it is recommended for use in the NHS (24).

In conclusion, PAS are pricing agreements proposed by pharmaceutical companies to enable patients to access high-value drugs. MAAs are data collection protocols added to CAAs (25). In 2016 the NHS introduced CAAs and MAAs, both of which are simpler compared to the complex PAS process (26).

### Some alternative indicators were used as clinical trial evidence

Except for overall survival/OS and progression-free survival/PFS, some other indicators such as progression-free survival/PFS, objective response rate/ORR, complete response/CR, relapse-free survival/RFS, disease-free survival/DFS, event-free survival /EFS, overall remission rate/ORR, minimal residual disease/MRD, response rates/RR, duration of response/DoR, time to next treatment, time to response were also used to evaluate the clinical outcome of drugs. Even though overall survival remains/OS (27) and progression-free survival/PFS (28) are the gold standard and commonly used outcomes for drugs, it has been proved that many oncology drugs do not provide benefits of PFS and OS (29). Therefore, along with the requirements for drug accelerated and medical reimbursement, more and more alternative indicators were used to evaluate the clinical outcomes, which were also applied to the complexity and specificity of orphan diseases.

### Potential association among recommendation, ICER/QALY value, and end of life

National Institute for Health and Care Excellence (NICE) typically defines a price of £20,000 to £30,000 per unit of QALY as cost-effective (30–32). In our study, the percentage of ICER/QALY values above £30,000 (53.3%) was higher than the percentage of ICER/QALY values below £30,000 except for drugs appraised in 2018. This suggests that the threshold ICER/QALY values are increasing to some degree.

The guide to the methods of technology appraisal had been published in 2013, and the purpose of the guide is to ensure that all interests should be considered when evaluating a treatment designed to prolong life. The guidance details that a “life-extending treatment at the end of life” treatment should satisfy two criteria (i) the treatment is indicated for patients with a short life expectancy, normally < 24 months, and (ii) there is sufficient evidence to indicate that the treatment has the prospect of offering an extension to life, normally of a mean value of at least an additional 3 months, compared with current NHS treatment. Besides, they should satisfy the two criteria and the evidence should be sufficient, and the assumptions used in the reference case economic modeling should be plausible, objective, and robust (33), if it has been considered as a drug that is a life-extending treatment, it would be given greater weight to QALYs achieved in the later stages of terminal diseases.

**Figure 3:**
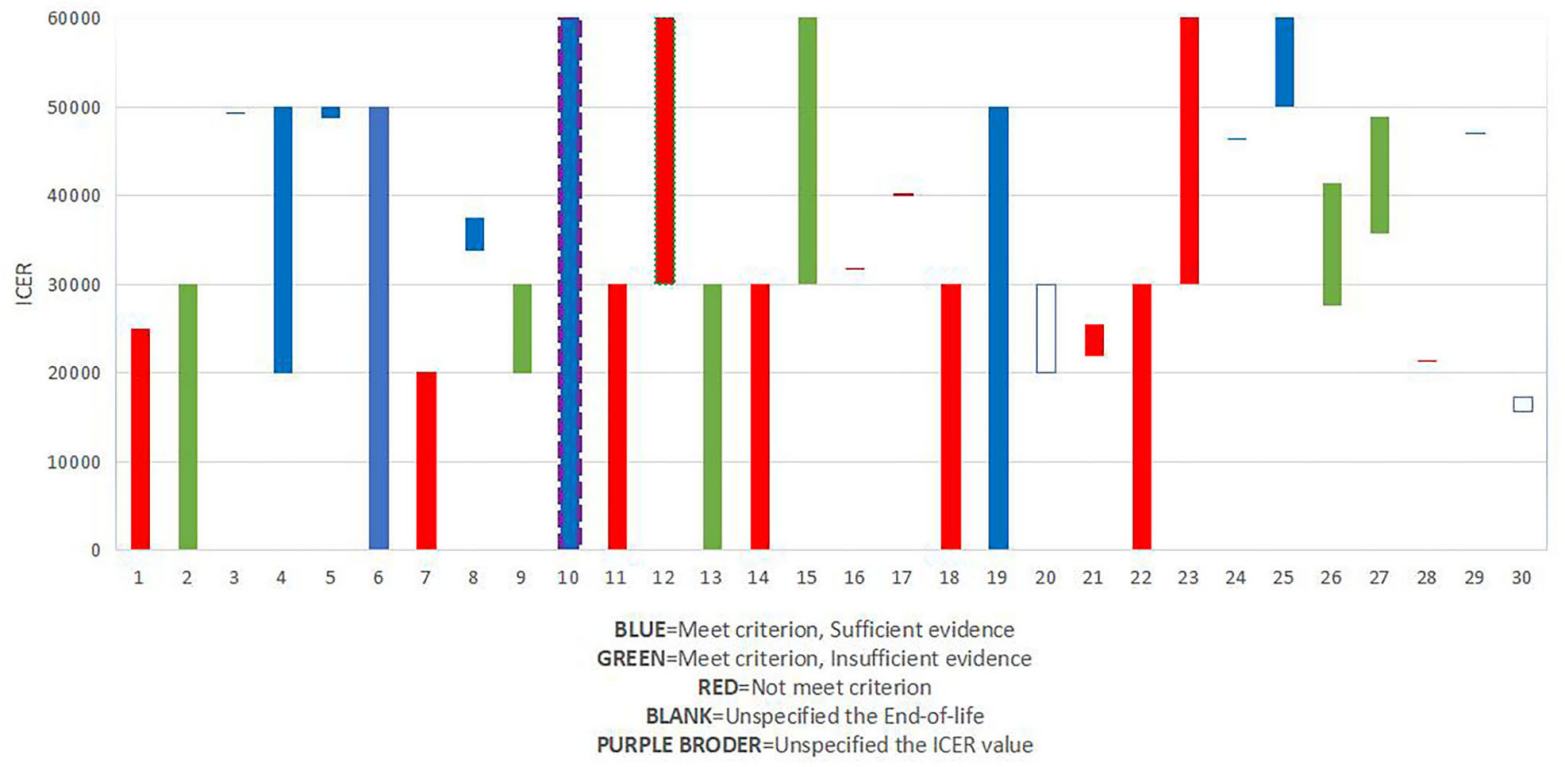
Relationship between end of life and ICER of orphan oncology drugs.

Besides, we explored the association between recommendation, ICER/QALY value, and end-of-life (Figure 3). Results showed that of all the 10 drugs that met the criterion of end of life and had sufficient evidence, 2 drugs were given a threshold of more than £50,000, and 8 drugs were given a threshold between £30,000 to £50,000, which might prove the importance of “end-of-life.”

### Drug delivery and other potential health-related benefits would be a new concern

Of all the 10 oral medications, 8 drugs (80%) were recommended to the NHS. This rate of recommendation was higher than that of drugs that needed to be administered *via* intravenous infusion. NICE takes into account not only the economics and effectiveness of the medication, but also the characteristics of the drug itself such as its delivery to patients, and it proved that some hidden factors had been given increasing attention, which would give more convenience for patients and their family, reduce non-medical costs and enhance the quality of life.

## Conclusion

The selection and appraisal process of NICE for orphan oncology drugs is important and also provides a good reference for other decision-makers. Attribute to the high treatment cost, small population, insufficient evidence of orphan oncology drugs, in order to solve the problem of a small number of subjects for validated trials and the uncertainty of clinical outcomes related to drug treatment, alternative indicators, special treatment of incomplete data, and appropriate economic models and HTA methods were used to estimate cost-effectiveness. Clinical evidence and cost-effectiveness are the basis of NICE's appraisal of orphan oncology drugs, but they also take into account factors such as the potential health-related benefits, its life-extending effects, and the impact of the medication on patient quality of life. Within the limits of affordable national medical insurance, how to improve the availability and accessibility of orphan oncology drugs became a global problem instead of a matter for any individual country, researchers should do their best to maximize the usage of orphan oncology drugs within limited resources.

## Data availability statement

The original contributions presented in the study are included in the article/supplementary material, further inquiries can be directed to the corresponding author.

## Author contributions

DS, LZ, and ZN were responsible for data collection and analysis. ZW, YY, LJ, and YN were responsible for data collection. All authors contributed to the article and approved the submitted version.

## Conflict of interest

The authors declare that the research was conducted in the absence of any commercial or financial relationships that could be construed as a potential conflict of interest.

## Publisher's note

All claims expressed in this article are solely those of the authors and do not necessarily represent those of their affiliated organizations, or those of the publisher, the editors and the reviewers. Any product that may be evaluated in this article, or claim that may be made by its manufacturer, is not guaranteed or endorsed by the publisher.
